# By cyclists, for cyclists: Road grade and elevation estimation from crowd-sourced fitness application data

**DOI:** 10.1371/journal.pone.0295027

**Published:** 2023-12-20

**Authors:** Elmira Berjisian, Alexander Bigazzi, Hamed Barkh

**Affiliations:** Department of Civil Engineering, University of British Columbia, Vancouver, British Columbia, Canada; Tsinghua University, CHINA

## Abstract

Road grade or slope is a key factor for walking and cycling behavior and outcomes (influencing route, speed, energy, etc.). For this reason, the scarcity of precise road grade data presents a challenge for travel information and analysis. This paper examines the accuracy of using crowd-sourced GPS data from a fitness application to estimate roadway grade profiles, which can then be used to develop network-wide road grade datasets. We externally validate an elevation estimation method described by McKenzie and Janowicz using field surveying data, and then propose and evaluate modifications for estimation of road grade (which is more directly relevant than elevation for walking and cycling analysis). We find that a modest amount of crowd-sourced GPS data can be used to generate relatively accurate road grade estimates: better than commonly-used low-resolution elevation models, but not as accurate as high-resolution data derived from LiDAR (Light Detection and Ranging). We also find that the grade estimates are more reliable than the elevation estimates, relative to alternative data sources. The most accurate method to aggregate crowd-sourced GPS data builds a composite roadway grade profile using partition-around-medoid clustering of individual grade sequences, first smoothed with a Savitzky-Golay filter and cleaned with Density-Based Spatial Clustering of Applications with Noise (DBSCAN). Implementing this method with an average of 150 GPS traces per location yields a root mean square error (RMSE) of 1% road grade. The findings in this paper can be used to incorporate precise road grade information into street network datasets over a wide spatial scale, which is necessary for walking and cycling analysis that fully considers the physiological aspects of active transportation.

## Introduction

The development of evidence-based strategies to promote active transportation (primarily walking and cycling) requires analysis of real-world active travel behavior. Fortunately, the widespread use of smartphones enables high-resolution spatial and temporal data collection from a large number of active travelers with a relatively low burden [1]. These rich crowd-sourced data are valuable in various types of walking and cycling analyses such as estimating volumes [2,3], studying air pollution exposure [4], identifying network improvements [5,6], and studying routes, accessibility, and traffic flow [7–9].

Road grade or slope (longitudinal changes in elevation over a given distance) is a main determinant of the effort or energy used in active travel, and so is a key factor influencing walking and cycling behaviors (speeds, routes, etc.) and outcomes (travel time, energy expenditure, pollution inhalation, etc.) [10–16]. Unfortunately, street network datasets usually lack elevation information [17], forcing researchers, analysts, and the traveler information industry (e.g., mapping and fitness applications) to rely on other data sources for roadway grade. Digital Elevation Models (DEM) and Digital Surface Models (DSM) are commonly used to estimate road grade, but their coarse resolution can result in poor road grade representation, and they often neglect elevated roadway surfaces [18,19]. Other sources such as LiDAR (Light Detection and Ranging) cloud point data, vehicle trajectories (time-stamped sequences of positional data), field surveying, or geometric design databases can provide better information, but are costly to collect, not widely available, and/or require substantial processing [18,20–23].

Altitude information from GPS devices provides another source of elevation data, with relatively poor accuracy that can be partially compensated by repeated measurements [24,25]. Today, crowd-sourced GPS trajectories from fitness, navigation, and routing applications enable repeated measurements over large geographical areas at a lower cost than using probe vehicles. McKenzie and Janowicz [26] proposed a method to estimate roadway elevation profiles from crowd-sourced GPS data (sourced from a fitness application); their method uses the median elevation of GPS records closest to equidistant sampling points along each road segment. In this paper, we expand on that research by 1) externally validating the elevation estimation method of McKenzie and Janowicz with an independent crowd-sourced GPS dataset, in comparison to ground-truth data from field surveying, 2) proposing and evaluating an extension of the method to generate road grade information, which is more directly relevant for active travel analysis than elevation, and 3) proposing and evaluating modifications to the method for improved accuracy of grade inference. This paper contributes to the literature by reporting improved methods for deriving road grade information from crowd-sourced GPS data, and determining the accuracy in comparison to field surveying measurements and alternative road grade data sources.

## Literature review

Raster elevation models (DEM, DSM) are the most commonly used data source in cycling studies to estimate roadway elevation changes or grade [14,27–31]. DEM and DSM are widely and readily available, but have the key shortcoming that they generally represent surrounding terrain rather than the travelled guideway, which can create substantial bias in inferred road grades [18]. Note that the terminology for elevation models varies; in this paper, we use DEM to refer to a model of bare-earth elevation and DSM to refer to a model that includes above-ground built and natural features (such as structures and vegetation). DEM represent bare-ground elevation, and hence fail to reflect elevation on roadway structures. Furthermore, DEM and DSM raster cells are usually coarse relative to the width of roadways, and so even a surface model (which includes structures) often misrepresents roadway elevations.

Grond [30] addressed the limitation of raster-based models by manually correcting elevation profiles on bridges, capping the grade at +/- 10%. Chen et al. [20] appended DEM and DSM data to a simplified Open Street Map (OSM) network, with heuristic corrections based on comparing the DEM and DSM, road design standards for tunnels, and an excessive grade threshold of 7%. Avoiding elevation models, Boyko and Funkhouser [22] proposed a method to extract roadway elevation profiles from dense three-dimensional LiDAR cloud point data. El Masri and Bigazzi [18] compared spatial data sources for road grade in active travel analysis and concluded that a high-resolution (0.5 m) DEM can generate reliable road grade information for non-elevated structures, whereas LiDAR cloud point data (processed using the Boyko and Funkhouser method) are needed for road grade information on elevated structures.

As an alternative to these areal spatial datasets, roadway elevation information can be derived by processing and aggregating probe vehicle trajectory data (typically, timestamped sequences of three-dimensional position records). Gupta et al. [32] aggregated probe vehicle data (acceleration, angular momentum, and speed) from 15 trips driven over a 9 km road segment to derive grade profiles based on a vehicle dynamic model with corrections from Google elevation data, achieving an accuracy of 0.5% grade compared to road inventory data. Fran et al. [33] used a fuel consumption model to estimate grade profiles from 90,000 sec of vehicle operating data, resulting in a root mean square error (RMSE) of 0.1% to 0.3% grade over a 4 km segment of mild-grade expressway.

GPS devices, such as included in most smartphones, can also provide traveler trajectory information, including altitude from satellite positioning, sometimes enhanced with barometric sensor data. Precision of moving horizontal position measurement for typical smartphone GPS data is 7 to 13 m [34], and vertical position error is around 1.5 to 3 times greater than horizontal position error [35,36]. A study comparing nine common GPS-based smartphone fitness applications reported errors in total elevation changes of 0 to 63 m over a 1 km running loop trip [37]. Meng et al. [24] used atmospheric pressure sensors to determine elevation changes, finding that barometry-based grade estimates (RMSE of 0.5% grade) were more accurate than estimates derived from GPS altitude information (RMSE of 2.6% grade). Another study comparing barometric altimeters reported standard errors of 1.5% to 1.9% in total elevation gain over a cycling trip using 28 different devices [38].

One approach to overcome the low precision of GPS-based altitude is to aggregate the information from many trajectories over the same roadway. John et al. [19] estimated elevation and grade from crowd-sourced GPS trajectories taken from OSM by averaging the values of nearby GPS trajectories, and evaluated against a high-resolution LiDAR-based DEM. Using 3,842 trajectories over 5,336 km of streets, they found an RMSE for elevation of 27 m and a mean grade error of 2.4%.

McKenzie and Janowicz [26] proposed a more sophisticated method to aggregate crowd-sourced GPS data into roadway elevation profiles. They first filter “efforts” (individual trajectories along a given roadway segment) to remove those with an insufficient number of GPS observations (no more than two standard deviations below the median number for the segment). A composite elevation profile is then created at 1-m intervals along the segment by aggregating from the most proximate elevation values in the filtered efforts (as the median or mean value at each 1-m node). Using a sample of 4 locations with 12,000 to 20,000 raw (unfiltered) efforts per location from Strava data, they report the accuracy of the method as RMSE of 1 to 5 m compared to LiDAR data. Accuracy for road grade inference was not investigated.

Beyond academic research, the traveler information industry also has an interest in providing road grade and elevation change data, particularly for mapping and fitness applications. For example, Google Maps, Strava, “Ride with GPS” (RwGPS), and others offer elevation profiles within their bicycle routing applications. RwGPS, which uses a low-resolution DEM, previously offered the option to manually correct elevations on elevated structures, and has recently reported improved estimation of elevation on such structures [39].

## Method

**Fig 1:**
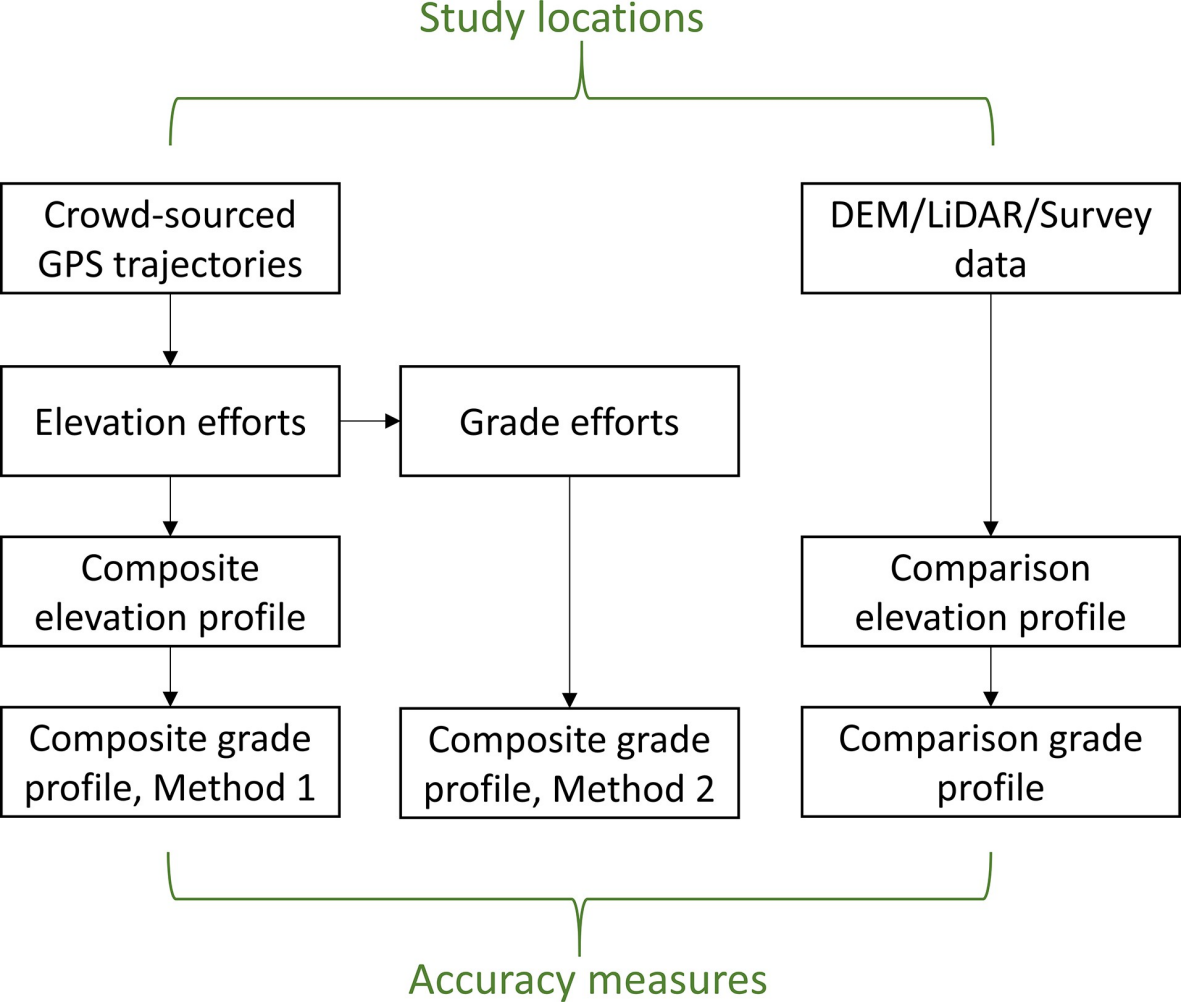
Methodology framework.

Fig 1 illustrates the study methodology. To accomplish the research objectives, we replicate the method described above from McKenzie and Janowicz [26] using an independent crowd-sourced dataset, and compare the results to several common alternative elevation data sources, as well as field surveying data. We also propose and compare the accuracy of modifications to the elevation estimation method (described below), as well as two methods of converting elevation information to road grade estimates.

### Study locations

Sixteen study locations (road segments) in the City of Vancouver were selected based on the availability of ground-truth field surveying data, popularity indicated by crowd-sourced volumes, and representation of a range of cycling facilities. Ground-truth field surveying data were available for 8 locations from a previous study [18]. The 8 most frequently used street segments in the crowd-sourced GPS data (see below) were identified for the other half of the study locations. Table 1 gives the sample locations and their elevation and grade attributes (data sources described below).

**Table 1:** Sample study locations.

Name	Length (m)	Elevated	Ground-truth survey data	Elevation (m)^1^	Grade (%)^1^
Burrard Bridge	1009	Yes	Yes	24.34 (5.80)	1.15 (2.71)
Main Street Bridge	253	Yes	Yes	9.72 (2.94)	-1.58 (6.07)
Cambie Ramp	129	Yes	Yes	8.38 (1.64)	4.36 (3.56)
West 10^th^ Street	119	No	Yes	29.26 (1.10)	-2.85 (0.84)
Beach Avenue	88	No	Yes	8.84 (0.08)	0.11 (0.60)
Island Park	73	No	Yes	4.22 (0.60)	-2.43 (3.19)
Smithe Street	73	No	Yes	18.96 (1.33)	-5.82 (0.79)
Trafalgar Street	58	No	Yes	28.40 (1.73)	-9.14 (1.49)
Cambie Bridge	338	Yes	No	17.40 (0.41)	0.04 (0.84)
Seaside	52	Yes	No	1.30 (0.00)	0.00 (0.00)
Adanac Street	173	No	No	13.75 (0.74)	-1.49 (0.18)
Stanley Park Drive	144	No	No	4.52 (0.09)	-0.24 (0.82)
False Creek	131	No	No	3.91 (0.25)	-0.58 (0.46)
Beach Street	123	No	No	8.86 (0.17)	0.50 (0.17)
Dunsmuir Street	100	No	No	28.43 (0.69)	2.15 (0.60)
Seawall	94	No	No	3.62 (0.31)	1.13 (0.58)

^1^ mean (standard deviation) at 1 m intervals.

### Crowd-sourced GPS data

We obtained crowd-sourced GPS trajectories from the now-retired fitness application Endomondo. Initially developed by Endomondo LLC, it was later acquired by UnderArmour Inc. The application was discontinued, along with its data interfaces on March 31, 2021. Before its termination, we gathered publicly available data for the Vancouver, Canada region in May 2020, by querying the Endomondo WorkoutAPI. The WorkoutAPI required two inputs: a UserID (a unique integer for each user) and a WorkoutID (with unique integers for each user, but not universally unique).

To identify relevant UserIDs, we sequentially submitted integers, starting from 1, to the Endomondo UserAPI, and then filtered for profiles with visibility set to “everyone” and with country set to Canada. We then queried WorkoutAPI for those UserIDs, only retaining those with activity timestamps using the Vancouver time zone (UTC offset of -7 during the summer months). Finally, we downloaded all summer (May through August) activities for these UserIDs from May 2010 through May 2020, yielding 27,052 raw trajectories in total. The activity files included the fields: UserID, WorkoutID, timestamp, latitude, longitude, altitude, distance, speed, duration, heart rate, sport (type of activity), and cadence.

The following rules were used to preprocess the trajectories, informed by the study objectives and past research using GPS data [40–43].

1. Discard trajectories outside metropolitan Vancouver, Canada.
2. Remove GPS records in the remaining trajectories that lack any of the essential attributes: timestamp, latitude, longitude, or altitude.
3. Identify duplicate timestamps in the same trajectory and retain the earliest one.
4. Reorder reverse time sequences.
5. Remove location errors based on “position jumps” where the distance traveled surpasses a 30 m buffer at a speed exceeding 50 m/s.
6. Split trajectories at a time gap greater than 8 hours.
7. Remove trajectories with fewer than 120 records.

A total of 9,031 trajectories remained after preprocessing, with a mean logging interval of 6.8 seconds (ranging from 1 to 67 seconds, standard deviation of 4.3 seconds). These GPS trajectories were map-matched using the PgMapMatch algorithm, developed by Miller-Ball et al. [44], with street network data extracted from OSM using overpassID [45]. PgMapMatch was selected based on recently demonstrated good performance for cycling GPS data [46]. We next identified the GPS trajectories that traversed any of our 16 study locations from the map-matched routes. For each of these trajectories, we extracted a sample effort (a subset of GPS records within the trajectory) using a 50-meter buffer around the study location. Finally, sample efforts were filtered based on the number of GPS records, consistent with [26], by removing any efforts with fewer than two standard deviations below the median number of records for efforts on that segment. The number of preprocessed and filtered efforts at each study location is given in Table 2. The filtered efforts and field surveying data for each location are given in the supporting information S1 Dataset.

**Table 2:** Number of crowd-sourced GPS traces (efforts) at each study location.

Study location	Preprocessed	Filtered	Cleaned (elevation)	Cleaned (grade)
Burrard Bridge	541	540	246	252
Main Street Bridge	21	21	10	0
Cambie Ramp	82	82	71	9
West 10^th^ Street	20	20	19	12
Beach Avenue	201	192	166	41
Island Park	179	176	162	59
Smithe Street	9	8	6	6
Trafalgar Street	12	11	11	10
Cambie Bridge	192	192	134	42
Seaside	365	329	327	222
Adanac Street	475	472	465	407
Stanley Park Drive	347	330	324	241
False Creek	379	374	356	193
Beach Street	528	507	481	382
Dunsmuir Street	400	393	376	273
Seawall	334	326	323	181

### Comparison elevation data sources

The accuracy of the crowd-sourced elevation and grade estimates are compared to several common elevation data sources:

1. A high-resolution (0.5 m grid) DEM for the City of Vancouver (CoVDEM) [47],
2. A low-resolution (20 m grid) DSM for Canada, the Canadian Digital Surface Model (CDSM) [48],
3. High-density LiDAR cloud point data for the City of Vancouver (CoVLiDAR) with a minimum density of 12 points per m^2^ [49],
4. Low-density LiDAR cloud point data covering the province of British Columbia (BCLiDAR) with a target density of 8 points per square meter [50], and
5. Route information from the smartphone application RwGPS.

Elevation profiles for the study locations were extracted from each data source at 1 m intervals along the segment. Elevation profiles were extracted from raster data (CoVDEM, CDSM) using bilinear interpolation. Elevation profiles were extracted from LiDAR data using the Boyko & Funkhouser method [22], which essentially fits a spline curve through the LiDAR cloud points. Elevation profiles were extracted from RwGPS by synthesizing the 1-m sampling points as a GPS trajectory and using the *route planner* and *replace elevation* features. Raw elevation data from the comparison data sources were smoothed using a Savitzky-Golay filter, consistent with previous studies [51,52]. Then road grade (in %) was calculated as changes in elevation at the same 1 m intervals along the elevation profile.

### Modification and extension to the McKenzie and Janowicz method

We propose and test several improvements to the crowd-sourced elevation estimation method described above from McKenzie and Janowicz [26], and extend its application to grade estimation. First, we smooth individual efforts (elevation profiles from a single GPS trajectory) using a Savitzky-Golay filter to reduce random error, as above. Second, we clean the efforts using Density-Based Spatial Clustering of Applications with Noise (DBSCAN) to mitigate systematic error [53]. DBSCAN groups the efforts into clusters and then labels any un-clustered efforts as Noise, which we then discarded.

DBSCAN requires two parameters: the minimum number of efforts needed to form a cluster (set as 2 in our case) and a cluster radius that determines how close efforts should be to each other to be part of the same cluster. For elevation, we set the cluster radius to account for the expected uncertainty in GPS-based vertical position. Assuming a Gaussian distribution in GPS error [54], we select 3 standard deviations as a threshold to capture the central 99.73% of the expected distribution of GPS records (i.e., excluding approximately 0.5% as outliers). The standard error of GPS-based vertical position is assumed to be around 15 m [34,54,55], leading to a cluster radius of 45 m for elevation. We set the cluster radius at 35% for grade, which is the maximum road grade in the world [22].

Our third modification involves testing two more sophisticated aggregation methods to create a composite profile from the filtered efforts, beyond the mean and median used in [26]: the clustering algorithms partition-around-medoid and kmlShape, both of which return an archetype (representative) effort for a given cluster of efforts. Partition-around-medoid is a clustering algorithm similar to k-means, but which uses an observed effort (medoid) as the archetype, in contrast to k-means in which the archetype is an average of the cluster [56]. KmlShape is a clustering algorithm that is based on the shape of efforts rather than a proximity measure (such as Euclidean distance used in partition-around-medoid and k-means) [57].

In addition to these three modifications (smoothing, cleaning, and aggregation method), we also evaluate two possible methods of converting from elevation to road grade estimates (illustrated in Fig 1). The first approach (Grade Method 1) involves calculating road grade from the composite elevation profiles, computed as the ratio of difference in elevation to the horizontal distance between sampling points along the segment (expressed in %). The second approach (Grade Method 2) is to create a composite road grade profile directly from individual grade efforts, with the rationale that the effect of intra-effort systematic bias in elevation may be reduced by working directly with grade values. Grade efforts are calculated from each elevation effort in the same way as Grade Method 1 (i.e., as the ratio of differenced elevation to the horizontal distance between GPS points along the segment, in %). A composite grade profile is then calculated from the filtered grade efforts using the same aggregation methods as the composite elevation profile.

Table 2 gives the number of preprocessed, filtered, and cleaned efforts at each study location. Note that no grade efforts remained for the Main Street Bridge location after cleaning, and so no Method 2 Grade estimates were generated for that location.

### Accuracy evaluation

Our modifications are tested by comparing the elevation and road grade accuracy of composite profiles created in six different ways:

1. Fmean: means of the filtered efforts,
2. Fmedian: medians of the filtered efforts (these are the original two methods used in [26]),
3. FSCmean: means of the filtered, smoothed, and cleaned efforts,
4. FSCmedian: medians of the filtered, smoothed, and cleaned efforts (these two show the effects of smoothing with Savitzky-Golay and cleaning with DBSCAN),
5. FSCpam: partition-around-medoid from the filtered, smoothed, and cleaned efforts, and
6. FSCkml: kmlShape from the filtered, smoothed, and cleaned efforts (these two show the effects of algorithmic aggregation rather than using mean or median).

For the eight locations with ground-truth data (see Table 1), estimated elevation and grade profiles are compared to profiles created directly from the field surveying measurements. For the eight locations without ground-truth data, the best available elevation data source is used as a ground-truth proxy, based on the findings in [18]: high-resolution (0.5 m) CoVDEM data for the non-elevated segments and high-density CoVLiDAR cloud-point data for the elevated segments. For internal validation, the ground-truth proxy measures are also compared to the ground-truth elevations for the eight locations with ground-truth data. Low-resolution comparison estimates are also created using low-resolution (20 m) CDSM data for the non-elevated segments and low-density BCLiDAR cloud-point data for the elevated segments.

Four evaluation measures are used to evaluate accuracy, in meters for elevation and meter/meter for grade (the first three were previously used in [26]):

1. Root mean squared error (RMSE) is the square root of the average squared difference between the profile values at the 1-m sampling points along each street segment,
2. Hausdorff distance (H) is the maximum distance between the profile values at the 1-m sampling points along each street segment,
3. Earth’s Mover distance (EM) compares two distributions in terms of the cost of converting one to the other, and
4. Frechet distance (F) is often explained as the shortest leash necessary to connect a dog to her owner as they walk along their respective trajectories, without going back on their trajectory.

Due to the high correlations among these measures, we use factor analysis to obtain an aggregate (unit-less) ‘Inaccuracy’ score that captures the maximum amount of common variance among the four evaluation measures [58]. The Inaccuracy score expression is obtained by the weighted sum score method [59] using Z-scores for each measure’s distribution across all profiles and locations, separately for elevation and grade.

To examine causes of inaccuracy, a set of contextual variables was created to represent environment and GPS trajectory characteristics, listed in Table 3, informed by the literature. Contextual land cover data were taken from the Metro Vancouver open data catalog (http://www.metrovancouver.org/data) and historical weather data from an online archive (https://www.timeanddate.com/weather/canada/vancouver/). Systematic associations between the contextual variables and Inaccuracy scores were investigated using Pearson correlation coefficients for continuous variables and t-tests of mean difference for binary variables. Finally, to evaluate the effect of the number of efforts on accuracy, a sensitivity test was conducted by estimating elevation and grade profiles using a randomly selected subset of 20%, 40%, 60%, and 80% of the filtered efforts at each location.

**Table 3:** Contextual variables used to examine sources of error.

Variable	Definition
Elevated	If the street segment is elevated, based on OSM tag “bridge” (binary)
Length	Length of the street segment in meters (continuous)
Tree cover	Proportion (by length) of street segments with tree canopy land cover intersecting (completely or partially) a 50-meter buffer around the sample segment (continuous)
Building cover	Proportion (by length) of street segments with building land cover intersecting (completely or partially) a 50-meter buffer around the sample segment (continuous)
Ground-truth comparison	If (non-proxy) ground-truth data from surveying are available for the segment (binary)
Elevation	Average elevation for the segment, in m (continuous)
Number preprocessed efforts	Number of preprocessed efforts available in the crowd-sourced dataset (continuous)
Number filtered efforts	Number of efforts retained after filtering (continuous)
Number cleaned efforts	Number of cleaned*efforts used to create the composite profile (continuous)
Logging interval	Average temporal difference between consecutive GPS points for filtered/cleaned efforts’ trajectories (continuous)
Speed outliers	Average difference between maximum and 95^th^ percentile speed, in m/s, for filtered/cleaned efforts’ trajectories (continuous)
Cloudy	Proportion of cloudy or rainy days when filtered/cleaned efforts occurred (continuous)
Mode	Whether the majority of filtered/cleaned efforts were recorded while cycling versus on foot (binary)

* Filtered, smoothed, and cleaned for FSCmean, FSCmedian, FSCpam, FSCkml; only filtered for Fmean and Fmedian.

## Results

### Inaccuracy score

As expected, RMSE, H, and F measures across composite elevation and grade profiles were highly correlated (Pearson correlation coefficients of at least 0.91); EM was less correlated with the other three measures (Pearson correlation coefficients ranging from 0.06 to 0.18). Factor analysis yields Eqs 1 and 2 for elevation and grade Inaccuracy scores, respectively, explaining 72%-75% of the variation among individual measures, with Kaiser–Meyer–Olkin (0.72–0.73) and Bartlett’s test of sphericity (p<0.01) confirming sampling adequacy and sufficient correlation between variables, respectively.


$$Inaccuracy_{El}=0.98\;Z_{RMSE}+0.99\;Z_{H}+0.24\;Z_{EM}+0.99\;Z_{F}\;[for\;Elevation]$$ (1)

$$Inaccuracy_{Gr}=0.96\;Z_{RMSE}+0.99\;Z_{H}+0.12\;Z_{EM}+0.99\;Z_{F}\;[for\;Grade]$$ (2)

### Accuracy of elevation and grade estimates

**Fig 2:**
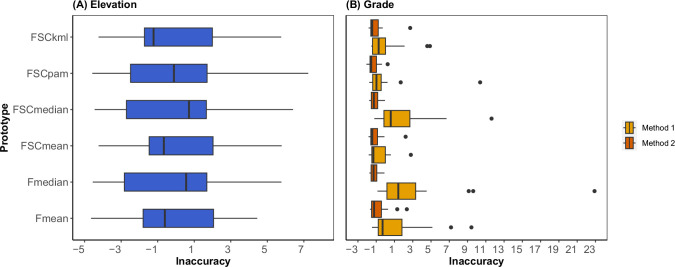
Elevation (A) and grade (B) Inaccuracy scores across 16 study locations for each profile generation method.

Fig 2 reports the distributions of Inaccuracy scores across locations for each profile generation method. More accurate methods produce lower-value (less inaccurate) distributions. Profile generation methods only produced significantly different Inaccuracy results (ANOVA test with p<0.05) for Method 1 grade estimates, for which FSCmean produced the most accurate grades followed by FSCpam. The aggregation method did not substantially impact the accuracy of elevation estimates, or grade estimates by Method 2. Between the two grade estimates, Method 2 produced consistently more accurate results.

The optimal profile generation methods, based on the lowest median Inaccuracy scores, are FSCkml for elevation, FSCmean for grade by Method 1, and FSCpam for grade by Method 2 (although, as noted above, the differences are not statistically significant for elevation or Method 2 grade). Table 4 gives the median (interquartile range) individual accuracy measures for these profiles across the 16 locations. Note that the accuracy measures for grade are expressed in % road grade, not percent errors. The grade profiles generated by Method 1 have about twice the inaccuracy of those generated by Method 2, as indicated by higher RMSE, H, and F.

**Table 4:** Median (interquartile range) of accuracy measures for optimal profile generation methods.

Accuracy measure	Elevation using FSCkml	Grade Method 1, using FSCmean	Grade Method 2, using FSCpam
RMSE	5.6 m (4.9 m)	2% (5%)	1% (3%)
H	5.8 m (5.6 m)	5% (10%)	2% (5%)
EM (unit-less)	0.1 (0.2)	0.3 (0.3)	0.3 (0.1)
F	5.8 m (5.6 m)	5% (10%)	2% (5%)

### Sources of error

Table 5 gives associations between contextual variables and Inaccuracy scores for the best-performing crowd-sourced data methods above (FSCkml for elevation, Method 2 with FSCpam for grade). Inaccuracy increased with fewer efforts available–particularly (and significantly) for grade estimates. The correlation magnitude between Inaccuracy and the number of efforts is greatest for preprocessed efforts, followed by filtered and then cleaned efforts. Consistent with this, grades were less accurate for the ground-truth locations, for which fewer efforts were available. Crowd-sourced grade Inaccuracy also significantly increased with lower GPS logging frequency and decreased for cycling activities (vs. foot travel), both of which lead to lower spatial density of records in each effort. Trajectories collected on cloudy days were also associated with higher Inaccuracy, consistent with previous findings that inclement weather degrades GPS location accuracy [60].

**Table 5:** Associations between crowd-sourced data Inaccuracy and contextual variables^1^.

Variable	Elevation	Grade
Elevated	0.88	-0.91
Length	-0.21	-0.24
Tree cover	0.08	0.21
Building cover	-0.23	-0.29
Ground-truth comparison	-0.18	3.82*
Elevation	-0.12	0.34
Number preprocessed efforts	-0.42	-0.73*
Number filtered efforts	-0.43	-0.71*
Number cleaned efforts	-0.32	-0.61*
Logging interval	-0.08	0.58*
Speed outliers	-0.23	0.39
Cloudy	0.52*	0.63*
Mode (cycling vs. foot)	-1.00	-3.38*

^1^ Pearson correlation for continuous variables and mean difference for binary variables.

* significantly different from zero at p<0.05.

### Effects of sample size (number of efforts)

Based on Table 1, filtering removes 3% of preprocessed efforts (averaged across locations), while cleaning removes another 14% of efforts for elevation and 47% of efforts for grade. The proportion of efforts removed by cleaning was higher for the 8 ground-truth locations (which already had fewer preprocessed efforts) than the 8 non-ground-truth locations: 57% versus 36% removed by cleaning for grade, respectively, and 21% versus 6% removed by cleaning for elevation.

**Fig 3:**
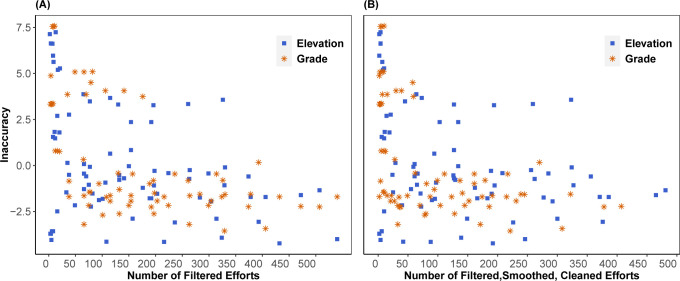
Inaccuracy score versus number of crowd-sourced filtered (A) and cleaned (B) efforts, using 20%, 40%, 60%, 80%, and 100% of available filtered efforts at each location.

Fig 3 shows Inaccuracy scores (again for the best-performing crowd-sourced data methods: FSCkml for elevation and FSCpam for grade Method 2) versus the number of filtered (left) and cleaned (right) efforts at each location, varying sample sizes by randomly retaining 20%, 40%, 60%, 80%, and 100% of the available filtered efforts at each location. The figure suggests a downward trend in Inaccuracy (i.e., improved accuracy) with both number of filtered and number of cleaned efforts, consistent with Table 5. However, the variation in Inaccuracy is wide, and the minimum Inaccuracy for each location is not always obtained with the most (100%) efforts. The risk of highly inaccurate crowd-sourced estimates appears to increase below around 200 filtered efforts, or 150 cleaned efforts, which could be considered a minimum threshold to use for elevation and grade estimates from crowd-sourced data. For a more quantitative estimate, we looked for local minima by fitting 4^th^-order polynomials to the data presented in each panel of Fig 3. The fitted curve for the filtered efforts has no real roots, but the fitted curve for the cleaned efforts (*R*^2^ = 0.24) has a local minim at 134 cleaned efforts. This threshold scales to 180 filtered efforts based on the linear relationship between the number of filtered and cleaned efforts at each location. The qualitative thresholds of 150 cleaned or 200 filtered efforts are similar but slightly more conservative.

### Comparison to other elevation data sources

**Fig 4:**
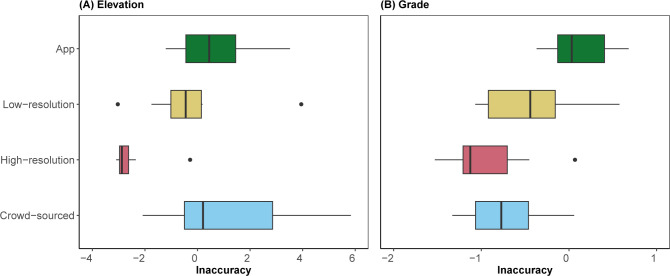
Elevation (A) and grade (B) Inaccuracy scores of different elevation sources for segments with ground-truth data. Note: for legibility, horizontal scale excludes a high Inaccuracy outlier value for App results.

Fig 4 compares Inaccuracy scores among elevation and grade data sources: the optimal crowd-sourced profiles above (using Method 2 for grade), high-resolution sources (CoVDEM and CoVLiDAR), low-resolution sources (CDSM and BCLiDAR), and the RwGPS application. Z-scores for these Inaccuracy values (Eqs 1–2) were recomputed using all four data sources, limited to the eight study locations with ground-truth data. High-resolution DEM/LiDAR data are the most accurate source of elevation and grade data, and RwGPS is the least accurate (based on median value). Crowd-sourced grade estimates outperform low-resolution DEM/LiDAR data, but crowd-sourced elevation estimates do not.

Median RMSE for crowd-sourced, high-resolution, low-resolution, and RwGPS data sources are 4.6, 0.3, 3.2, and 4.7 m, respectively, for elevation and 4%, 1%, 5%, and 8%, respectively, for road grade. Note that the eight ground-truth locations are poorer-performing for crowd-sourced grade data than the other locations because they had fewer efforts available. The ground-truth locations averaged 86 and 49 cleaned efforts for elevation and grade estimates, respectively, while the non-ground-truth locations averaged 348 and 243 (Table 1). Hence, this comparison is conservative in presenting the relative accuracy of smaller-sample crowd-sourced data.

Comparing our elevation estimation results to McKenzie and Janowicz [26], they found RMSE ranging from 1.2 to 5.0 m across 4 locations, whereas we find similar but slightly poorer performance using the same method on our dataset, with a median RMSE of 6.7 m using Fmedian to create composite profiles. The small performance difference could be due to several factors. Firstly, [26] only examined non-elevated road segments (which tend to have smaller elevation error), whereas 5 of our 16 study locations were elevated. In terms of data, [26] utilized a larger number of efforts per location, which also reduces elevation error. In addition, the studies used different GPS data sources (Endomondo vs. Strava in [26]), and these applications might employ different (unpublished) pre-processing steps affecting the “raw” elevation data [37]. Methodologically, [26] applied an additional filter for devices with barometric altimeters, which might have restricted their dataset to higher-precision GPS records (this could not be implemented in our study due to a lack of device data in Endomondo). Finally, [26] measured accuracy compared to LiDAR data, whereas we compare to both LiDAR and field surveying data (although we did not find that a survey-based reference source degraded elevation accuracy).

Comparing our results to the crowd-sourced GPS method in [19] we find improved results for elevation (they report an RMSE of 27 m) and for grade (they report a mean error of 2.4% road grade). Trajectory-based methods using probe vehicle sensor data [32,33] have reported greater accuracy (RMSE of 0.1% to 0.5% road grade), but rely on additional sensors that are not available in crowd-sourced GPS data.

We can contextualize these results using the relationship between road grade and energy expenditure, since energy or effort is a primary mediator of cycling behavior and outcomes (as stated above). Using typical urban cyclist physical parameters, cycling power *P* in watts can be estimated as

$$P=1030(0.0077+G)v+0.342v^{3}$$ (3)

where *G* is the road grade (unit-less) and *v* is the cycling speed in m/s [61]. Assuming a 20 km/hr (5.56 m/s) cycling speed on level ground [62], even an error of 1% road grade (*G*±0.01) leads to a 57 W (56%) error in calculated cycling effort. Supporting this, in cycling route choice research, a 1% road grade can be considered “steep”, and a 3.5% grade “very steep” [63]. Thus, accuracy differences of 1% road grade are physically and behaviorally meaningful in cycling analysis.

## Conclusion

The results demonstrate that crowd-sourced GPS data, even in modest amounts, can provide relatively accurate roadway grade estimates–better than the commonly-used low-resolution DEM, but not as good as high-resolution DSM or cloud-point LiDAR data. Our external validation of the elevation estimation method proposed by McKenzie & Janowicz [26] with field surveying data supports the reliability of the method, even on elevated structures. We have also demonstrated small accuracy improvements from several enhancements to the method, including smoothing, cleaning, and using a clustering algorithm to aggregate efforts into a composite profile. And we determined that the most accurate way to extract grade data from crowd-sourced GPS is to aggregate individual grade profiles directly, rather than calculating grade from a composite elevation profile (which yields twice the error).

A related important finding is that although roadway elevation and grade are closely related, methodological findings for one cannot be directly transferred to the other. The accuracy of crowd-sourced grade estimates improves with the amount and resolution of GPS data available, among other factors, whereas the accuracy of crowd-sourced elevation estimates was shown to be less sensitive to these data attributes. Compared to other data sources, crowd-sourced grade estimates are more competitive than crowd-sourced elevation estimates, out-performing low-resolution DEM/LiDAR data for grade but not for elevation estimates. High-resolution DEM/LiDAR data still proved to be the most accurate across all locations, and should be used wherever available.

We recommend that future work to develop network-wide road grade data (such as for integrating with OSM) from crowd-sourced GPS data use a partition-around-medoid clustering algorithm to aggregate individual grade efforts, after applying a Savitzky-Golay filter for smoothing and DBSCAN for cleaning, which provides a RMSE in road grade of around 1%. Our study suggests that a minimum sample of approximately 200 GPS traces (prior to cleaning) would serve as a reasonable threshold for crowd-soured elevation and grade estimates. More accurate road grades can be extracted from cloud-point LiDAR data, where they are available in densities sufficient for road grade estimation. Unfortunately, due to costs LiDAR data are inconsistently available, even in a region as populated as metropolitan Vancouver, Canada. Additionally, LiDAR data are often collected in urban areas, creating inequities in data quality for rural active travelers. Thus, crowd-sourced GPS data provide the opportunity to derive road grades for the complete street network.

We also recommend additional research to further validate the proposed grade estimation method with independent datasets in other locations. Without external validation, we cannot say with certainty that the accuracy improvements we observed are transferable to other datasets (e.g., those that are larger or generated from different applications or devices). Further investigations are also needed into the impacts of varying sources of trajectory data and DBSCAN parameters. Qualitatively, smaller/narrower thresholds for the DBSCAN cluster radius result in more trajectories being removed through cleaning, with a trade-off of fewer but higher-quality trajectories remaining for inference. This effect can be seen in the difference in our results between estimates using cleaned data (using our DBSCAN parameters) versus only filtered data (which larger/wider DBSCAN cluster radii approach at the limit). For example, at the Burrard Bridge study location all 540 filtered efforts are identified as valid with a very large DBSCAN cluster radius, whereas the 45 m radius removes 294 efforts through cleaning. Varying the threshold ±15 m results in 335 efforts removed at a 30 m radius and 224 efforts removed at a 60 m radius. Further research is needed to determine the optimal DBSCAN parameters in varying contexts, potentially contingent on dataset attributes (size, GPS device types, temporal resolution, application pre-processing, etc.), segment attributes (length, grade, elevated or not, surrounding terrain and land use, etc.), and other factors.

Finally, we reiterate the suggestion in [26] that continued efforts be made to incorporate (precise) road grade information into publicly-available street network datasets such as OSM. Accurate road grade data are crucial for analysis of active travel behavior (speed, route choices) and outcomes (travel time, energy expenditure), and the current lack of reliable data impedes analysis that fully considers the physiological aspects of active travel.

## Supporting information

S1 Dataset The complete set of three-dimensional GPS and surveying data used in the analysis. (XLSX)
